# Fetal karyotyping in adolescent pregnancies: a population-based cohort study on outcomes of invasive prenatal testing

**DOI:** 10.3389/fgene.2025.1581249

**Published:** 2025-04-25

**Authors:** Jakub Staniczek, Maisa Manasar-Dyrbuś, Patrycja Sodowska, Krzysztof Sodowski, Agata Włoch, Bartosz Czuba, Wojciech Cnota, Monika Paul-Samojedny, Agnieszka Kania, Henryka Sodowska, Magda Rybak-Krzyszkowska, Adrianna Kondracka, Rafał Stojko, Agnieszka Drosdzol-Cop

**Affiliations:** ^1^ Chair and Department of Gynecology, Obstetrics and Gynecological Oncology, Medical University of Silesia, Katowice, Poland; ^2^ Department of Gynecology, Obstetrics, Gynecological Oncology, Pediatric and Adolescent Gynecology, Bonifraters’ Medical Center, Katowice, Poland; ^3^ Genom (Godula Hope) Medical Center, Ruda Śląska, Poland; ^4^ Sodowscy Medical Center, Katowice, Poland; ^5^ Chair and Department of Gynecology and Obstetrics, Medical University of Silesia, Ruda Śląska, Poland; ^6^ Department of Medical Genetics, Medical University of Silesia, Sosnowiec, Poland; ^7^ Department of Obstetrics and Perinatology, University Hospital, Krakow, Poland; ^8^ Department of Obstetrics and Pathology of Pregnancy, Medical University of Lublin, Lublin, Poland

**Keywords:** adolescent pregnancy, karyotyping, invasive procedures, amniocentesis, CVS

## Abstract

**Background:**

Adolescent pregnancies present unique challenges in prenatal diagnostics, yet data on the prevalence and types of chromosomal abnormalities in this population remain limited.

**Objective:**

This study aimed to assess the prevalence and spectrum of chromosomal abnormalities and evaluate the effectiveness of invasive prenatal diagnostic procedures.

**Methods:**

A retrospective cohort study analyzed data from invasive prenatal diagnostic procedures (amniocentesis and transabdominal chorionic villus sampling) and fetal karyotyping in adolescent pregnancies, comparing them with data obtained from pregnancies in older women.

**Results:**

Abnormal karyotype prevalence varied by age. Trisomies were least frequent in adolescents (5.9%) vs. women 20–34 (9.3%) and ≥35 years (12.1%). Turner syndrome was more common in adolescents (4.6%) than in women 20–34 (2.8%) or ≥35 years (0.1%). Adolescents had a higher risk of unspecified fetal sex (RR = 2.25, 95% CI: 1.16–4.35) and culture failure (RR = 4.32, 95% CI: 2.07–9.00). Ultrasound abnormalities were the main reason for invasive testing (86.3%, p < 0.001). More chorionic villus sampling procedures were needed per abnormal karyotype in adolescents (3.25) vs. women 20–34 (2.42) or ≥35 years (2.19), while fewer amniocenteses were required (6.68 vs. 7.37 and 8.44).

**Conclusion:**

Adolescents show unique chromosomal abnormalities, underscoring the need for tailored prenatal counseling and diagnostics.

## Introduction

### Background

Invasive prenatal diagnostic procedures and fetal karyotyping are invaluable tools for evidence-based management of fetal abnormalities in high-risk pregnancies (Chang et al., 2012; Jummaat et al., 2019). Amniocentesis and chorionic villus sampling (CVS) are crucial for detecting chromosomal abnormalities in fetuses early (Alfirevic et al., 2017; Zhu et al., 2016).

Conventional karyotyping remains a cornerstone of prenatal genetic diagnosis. It reliably identifies significant chromosomal anomalies in pregnancies with fetal ultrasound abnormalities and has been the gold standard for decades, providing critical diagnostic information. During the study period, this classical karyotyping approach was indeed the gold standard in Poland (partly because it was reimbursed by the National Health Fund, NFZ). However, recent international and national guidelines (e.g., The American College of Obstetricians and Gynecologists, The Polish Society of Gynecologists and Obstetricians) now recommend chromosomal microarray analysis (array CGH) as the preferred first-line test for fetuses with structural anomalies, reflecting an evolution from classical to molecular karyotyping (Committee Opinion No, 2016; Sieroszewski et al., 2022). Despite significant advancements in molecular diagnostic, karyotyping has remained fundamental in prenatal diagnosis and genetic counseling, offering insights into fetal chromosomal health (Eren Keskin et al., 2024; Lichtenbelt et al., 2011; Moczulska et al., 2023).

The impact of advanced maternal age on fetal karyotypic abnormalities is well-documented; however, the influence of adolescent pregnancy remains insufficiently studied. Women of advanced maternal age (≥35 years) exhibit a significantly increased incidence of chromosomal aneuploidies, including autosomal trisomies and sex chromosome aneuploidies (Cuckle and Morris, 2020; Li et al., 2021; Warburton et al., 2016). Human female meiosis is inherently error-prone (Hassold and Hunt, 2009). Most autosomal trisomies result from nondisjunction events occurring during the first maternal meiotic division, with maternal age being a key determinant of these errors (Hassold et al., 2007).

Studies of women of advanced maternal age have reported a higher prevalence of trisomy 21 and other chromosomal abnormalities, as demonstrated by Kim et al., (2013). In adolescent pregnancies, the overall risk of common aneuploidies has traditionally been considered lower compared to pregnancies in women over 35 years old. However, emerging evidence suggests that specific chromosomal abnormalities may be more prevalent in this younger cohort (Staniczek et al., 2024). Significantly, the risk does not decrease linearly with decreasing maternal age, and specific fetal karyotypic abnormalities may not follow the conventional age-related pattern. Recognizing these differences is essential for understanding the etiological factors contributing to chromosomal abnormalities in adolescent pregnancies (Li et al., 2021; Drosdzol-Cop et al., 2023).

A review of the literature revealed no prior studies specifically addressing the use of invasive prenatal testing in adolescent pregnancies and karyotypic evaluation of fetuses in this cohort. This study, conducted within a Polish cohort, aims to fill this gap and provide novel insights into chromosomal abnormalities detected in adolescent pregnancies. Notably, while advanced maternal age is widely recognized as a risk factor for aneuploidy, it is not the sole determinant, emphasizing the need for a more comprehensive understanding of genetic risks in younger groups. These findings underscore the necessity for further research to optimize prenatal care strategies for adolescent pregnancies.

### Objectives

This study aims to assess the prevalence and spectrum of chromosomal abnormalities and evaluate the effectiveness of invasive prenatal diagnostic procedures in pregnancies stratified by maternal age groups. The analysis additionally focuses on the clinical indications for invasive testing, the types of procedures performed, and their distribution and timing relative to maternal age.

## Methodology

### Study design

A retrospective cohort study analyzed data from invasive prenatal diagnostic procedures (amniocentesis and transabdominal chorionic villus sampling) and fetal karyotyping in adolescent pregnancies, comparing them with data obtained from pregnancies in older women. The study was approved by the Review Board of the Chair and Department of Gynecology, Obstetrics, and Gynecological Oncology at the Medical University of Silesia, Katowice. The Bioethics Committee of the Medical University of Silesia, Katowice, Poland waived the need for ethics approval and the need to obtain consent for the collection, analysis and publication of the retrospectively obtained and anonymized data for this non-interventional study. The study was conducted in accordance with ethical principles governing medical research, including the Declaration of Helsinki. All personal data were securely protected and remained confidential within the participating research center. The study’s design and implementation followed the STROBE (Strengthening the Reporting of Observational Studies in Epidemiology) guidelines, ensuring a rigorous and reproducible research methodology (von Elm et al., 2008).

### Settings

From 1 January 2004, to 30 November 2024, all data from patients meeting the study’s inclusion criteria were included. The study was conducted in the Silesian Voivodeship of Poland, with data gathered from the following prenatal care centers: the Sodowski Medical Center in Katowice, the Genom (Godula Hope) Medical Center in Ruda Śląska and the Department of Gynecology, Obstetrics, Gynecological Oncology, Pediatric and Adolescent Gynecology in Katowice. The data were then analyzed by the Chair and Department of Gynecology, Obstetrics, and Oncological Gynecology team at the Medical University of Silesia, Katowice.

### Participants

The study included participants with singleton pregnancies who underwent invasive prenatal diagnostic procedures (amniocentesis or transabdominal chorionic villus sampling) and routine G-banded karyotyping, with a minimum of 20 metaphase cells analyzed at a resolution of at least 550 bands. Patients who underwent alternative cytogenetic analyses, such as array-based comparative genomic hybridization (aCGH) or fluorescence *in situ* hybridization (FISH), were excluded. Additionally, participants with incomplete medical records were not included in the study.

The study population was divided into two primary groups: adolescent pregnancies (women aged 19 or younger) and a control group comprising all other pregnant women. The control group was further stratified into two age categories: 20–34 and ≥35.

### Variables

This analysis identified quantitative (age, parity, and gestational week at the time of examination) and qualitative (indication for invasive procedures, type of invasive procedure, fetal sex, karyotypes, and kind of aberration) variables. Age and gestational week were treated as continuous variables (expressed in years and weeks, respectively), while parity was reported using medians and ranges, given its discrete nature. Depending on whether the data followed a normal distribution, these variables were presented using measures of central tendency and dispersion (mean with standard deviation or median with interquartile ranges).

Five key categories were identified among the qualitative variables: the indication and type of invasive procedure, fetal sex, karyotypes presented across multiple categories (including expected results and specific abnormalities), and the kind of aberration, which offered further detail on chromosomal anomalies.

Fetal sex was classified as female, male, or “unspecified fetal sex” if the karyotype alone did not allow to clear sex assignment. The category of “unspecified fetal sex” was used when karyotyping could not definitively determine sex–for instance, due to insufficient sample quality, failed cell culture, or certain mosaic or sex chromosome abnormalities that precluded unambiguous sex determination. Furthermore, cases with sex chromosome aneuploidies were counted under “unspecified fetal sex” if the karyotype alone did not allow a clear sex assignment.

### Data sources

The dataset includes karyotype results stored using the Astraia software package from NEXUS/ASTRAIA GmbH. All patient data were anonymized and processed in compliance with relevant regulations. Five researchers independently analyzed and extracted anonymized data and adjusted the karyotype results according to ISCN, 2024 - An International System for Human Cytogenomic Nomenclature, (2024).

### Bias

Several potential sources of bias were identified, and specific strategies were implemented to mitigate their impact on our findings. All invasive procedures were performed by physicians specializing in Obstetrics and Gynecology or Perinatology (Maternal-Fetal Medicine), with extensive experience in invasive prenatal diagnostics. Additionally, cytogenetic diagnostics were conducted at the reference diagnostic laboratory of the Genom (Godula Hope) Medical Center in Ruda Śląska, Poland. This laboratory is registered in the laboratory registry maintained by the National Council of Laboratory Diagnosticians in Poland. It holds a certificate of recommendation from the Polish Society of Human Genetics.

To ensure the highest quality of data analysis and interpretation, the research team comprised experts from diverse fields: Obstetrics and Gynecology (J.S., M.M.-D., R.S., P.S., M.R.-K., A.K., K.S., B.C., W.C., A.D.-C.), Perinatology (Maternal-Fetal Medicine) (J.S., A.K., K.S., B.C., W.C.), Clinical Genetics (M.R.-K., A.K., H.S.), Laboratory Diagnostics (M.P.-S., A.K.), Pediatrics (A.W.), and Pediatric and Adolescent Gynecology (A.D.-C.).

To reduce selection bias, we included a comprehensive cohort of all pregnancies undergoing invasive procedures over 20 years in the Silesian Voivodeship in Poland. Moreover, because missing data can introduce bias and undermine the validity of study results, incomplete patient records were excluded to mitigate this risk further.

### Study size

The study was conducted with a sample of 8,155 participants, divided into three age groups to ensure representativeness and facilitate the analysis of demographic differences. The sample included 153 participants aged up to 19, 4,287 participants in the 20–34 age range, and 3,715 participants in the 35-year-old and older age group.

### Statistics

The results of the analysis are presented in tables. Quantitative variables with a normal distribution are expressed as the mean with standard deviation. In contrast, quantitative variables with a distribution significantly deviating from normality are presented using the median with the interquartile range. Distribution was assessed using quantile-quantile plots. For intergroup comparisons of qualitative variables, the Chi-square test was used. For comparisons of quantitative variables with a normal distribution, the Student’s t-test was applied, while for variables significantly deviating from a normal distribution, the Wilcoxon test or, in the case of multiple comparisons, the KruskalWallis test was used. Post hoc tests for significant pairwise comparisons are illustrated using boxplots. A p-value of <0.05 was considered statistically significant. Statistical analysis was performed using R software within the RStudio environment.

## Results

### Quantitative data analysis

The number of deliveries increases with maternal age, reflecting the natural course of fertility and reproductive preferences across age groups. In the ≤19 years group, nulliparous women predominate, whereas in the ≥35 years group, the highest median and mean number of deliveries are observed.

**FIGURE 1:**
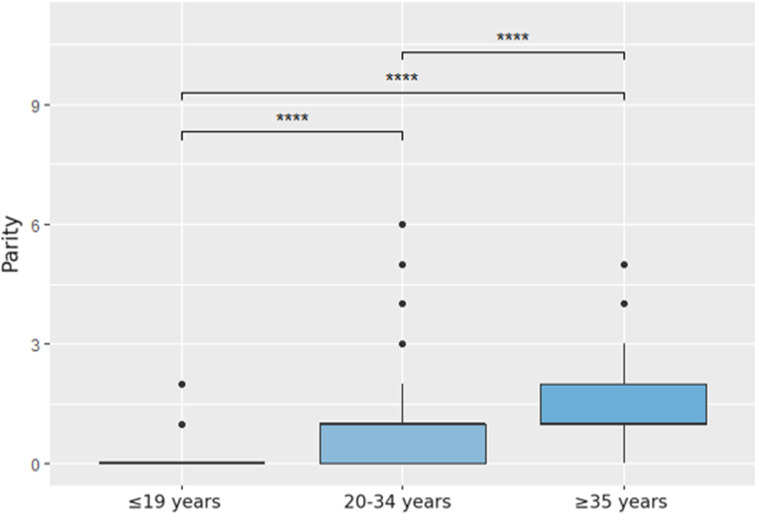
Comparison of parity by age group, *post hoc* analysis using the Wilcoxon test.

The gestational age at the time of the procedure varied across age groups, with a median of 16 weeks. However, the ≤19 years group had a slightly higher mean gestational age (18.16 weeks, SD = 4.53) compared to older groups (17.43 weeks in 20–34 years and 17.23 weeks in ≥35 years) (Table 1, Figure 1).

**TABLE 1:** Comparison of quantitative variables by age group–KruskalWallis test.

Group	n	Q1	Q3	Median	Mean	SD	p-value
Parity							
≤19 years	153	0	0	0	0,118	0,379	<0,001
20–34 years	4,287	0	1	1	0,637	0,697	
≥35 years	3,715	1	2	1	1,372	0,778	
Week of pregnancy during invasive procedure							
≤19 years	152	15	21	16	18,164	4,527	0,2871
20–34 years	4,287	15	20	16	17,435	3,901	
≥35 years	3,715	15	19	16	17,226	3,272	

SD, Standard Deviation.

### Qualitative data analysis

Participants were divided into three age groups: ≤19 years (153), 20–34 years (4,287), and ≥35 years (3,715, Table 2). The most frequent cytogenetic result in all groups was a normal karyotype (82.0%–84.5%). A statistically significant increase in the incidence of chromosomal trisomies (mainly Down, Edwards, and Patau syndromes) was observed with advancing maternal age (p < 0.001): 5.9% in the ≤19 age group, 9.3% in the 20–34 group, and 12.1% in the ≥35 group. A similar trend was seen for Down syndrome, rising from 2.6% among adolescents to 8.2% in the oldest group. Turner syndrome (45,X) was detected more frequently in adolescents (4.6%) than in older groups (2.8% and 0.1%, respectively).

**TABLE 2:** Summary of qualitative variables by age group.

Variable	≤19 years (n)	20–34 years (n)	≥35 years (n)	p-value
n	153	4,287	3,715	
Indication for invasive procedures (%)				
Abnormal fetal ultrasound result	132 (86,3)	3,287 (76,7)	2095 (56,4)	<0,001
Patient’s request	0 (0,0)	5 (0,1)	6 (0,2)	
Family history of genetic disorders	1 (0,7)	108 (2,5)	56 (1,5)	
High risk in the first-trimester combined screening test	20 (13,1)	887 (20,7)	1,289 (34,7)	
Patient’s age over 35 years	0 (0,0)	0 (0,0)	269 (7,2)	
Invasive procedures (%)				
CVS (%)	26 (17,0)	689 (16,1)	399 (10,7)	<0,001
Amniocestesis (%)	127 (83,0)	3,598 (83,9)	3,316 (89,3)	
Fetal sex (%)				
Female	72 (47,1)	1985 (46,3)	1744 (46,9)	<0,001
Male	72 (47,1)	2,190 (51,1)	1920 (51,7)	
Unspecified fetal sex	9 (5,9)	112 (2,6)	51 (1,4)	
Aberration (%)				
Normal karyotype	126 (82,4)	3,514 (82,0)	3,140 (84,5)	<0,001
Culture failed	8 (5,2)	87 (2,0)	45 (1,2)	
Common trisomies	9 (5,9)	399 (9,3)	451 (12,1)	
Other (Unbalanced translocation and other unbalance aberations)	2 (1,3)	59 (1,4)	50 (1,3)	
Polyploidy	1 (0,7)	56 (1,3)	5 (0,1)	
Sex chromosomal aneuploidies	7 (4,6)	146 (3,4)	16 (0,4)	
Balanced abberation	0 (0,0)	26 (0,6)	8 (0,2)	
Abnormal kariotypes (%)				
Balanced abberation	0 (0,0)	1 (0,0)	0 (0,0)	<0,001
Balanced Robertsonian translocation	0 (0,0)	2 (0,0)	1 (0,0)	
Balanced translocation	0 (0,0)	10 (0,2)	4 (0,1)	
Cat Eye Syndrome	1 (0,7)	2 (0,0)	0 (0,0)	
Down syndrome	4 (2,6)	225 (5,2)	303 (8,2)	
Down syndrome with pericentric inversion on 9	0 (0,0)	3 (0,1)	0 (0,0)	
Edwards syndrome	2 (1,3)	108 (2,5)	111 (3,0)	
Edwards syndrome with pericentric inversion 10	0 (0,0)	1 (0,0)	0 (0,0)	
Jacobs syndrome	0 (0,0)	3 (0,1)	2 (0,1)	
Klinefelter syndrome	0 (0,0)	5 (0,1)	2 (0,1)	
Maternally inherited balanced translocation	0 (0,0)	7 (0,2)	1 (0,0)	
Mosaic Down syndrome	0 (0,0)	5 (0,1)	5 (0,1)	
Mosaic Edwards syndrome	0 (0,0)	1 (0,0)	0 (0,0)	
Mosaic Turner syndrome	0 (0,0)	8 (0,2)	5 (0,1)	
Other structural unbalance	1 (0,7)	18 (0,4)	15 (0,4)	
Tetrasomy 18q syndrome	0 (0,0)	0 (0,0)	1 (0,0)	
Patau syndrome	3 (2,0)	45 (1,0)	31 (0,8)	
Patau syndrome with pericentric inversion on 9	0 (0,0)	2 (0,0)	0 (0,0)	
Paternally inherited balanced translocation	0 (0,0)	6 (0,1)	2 (0,1)	
Pericentric inversion on chromosome 10 (p11q21)	0 (0,0)	0 (0,0)	1 (0,0)	
Pericentric inversion on chromosome 2 (p11,2q13)	0 (0,0)	0 (0,0)	1 (0,0)	
Pericentric inversion on chromosome 5 (p13q13)	0 (0,0)	3 (0,1)	1 (0,0)	
Pericentric inversion on chromosome 9 (p11q12)	0 (0,0)	13 (0,3)	15 (0,4)	
Pericentric inversion on chromosome 9 (p12q13)	0 (0,0)	14 (0,3)	13 (0,3)	
Ring chromosome 13	0 (0,0)	1 (0,0)	0 (0,0)	
Translocation Down syndrome	0 (0,0)	4 (0,1)	1 (0,0)	
Translocation Patau syndrome	0 (0,0)	3 (0,1)	0 (0,0)	
Triple X syndrome	0 (0,0)	9 (0,2)	2 (0,1)	
Triploidy	1 (0,7)	56 (1,3)	5 (0,1)	
Trisomy 9 syndrome	0 (0,0)	3 (0,1)	1 (0,0)	
Turner syndrome	7 (4,6)	119 (2,8)	5 (0,1)	
Turner syndrome with a translocation between the X chromosome and chromosome 14	0 (0,0)	1 (0,0)	0 (0,0)	
Turner syndrome with pericentric inversion	0 (0,0)	1 (0,0)	0 (0,0)	
Unbalanced translocation	0 (0,0)	5 (0,1)	1 (0,0)	
Unbalanced translocation – mosaic Turner syndrome with ring chromosome 14	0 (0,0)	1 (0,0)	0 (0,0)	
Unbalanced translocation between chromosomes 5 and 14 with monosomy 14	0 (0,0)	1 (0,0)	0 (0,0)	
Wolf-Hirschhorn syndrome	0 (0,0)	0 (0,0)	1 (0,0)	
Detailed abnormal karyotypes (%)				
mos 45,X,[1]/46,XX[3]	0 (0,0)	1 (0,0)	0 (0,0)	<0,001
mos 45,X,der(14)t(X; 14) (q10; q10)[9]/45,X	0 (0,0)	1 (0,0)	0 (0,0)	
mos 45,X[10]/46,XX[10]	0 (0,0)	1 (0,0)	0 (0,0)	
mos 45,X[10]/46,XX[4]	0 (0,0)	1 (0,0)	0 (0,0)	
mos 45,X[116]/46X,+mar[18]	0 (0,0)	1 (0,0)	0 (0,0)	
mos 45,X[15]/46,XX[35]	0 (0,0)	0 (0,0)	1 (0,0)	
mos 45,X[4]/4.6.46,XX[26]	0 (0,0)	0 (0,0)	1 (0,0)	
mos 45,X[4]/46,XX[26]	0 (0,0)	0 (0,0)	2 (0,1)	
mos 45,X[4]/46,XX[8]	0 (0,0)	1 (0,0)	0 (0,0)	
mos 45,X[6]/46,XX[11]	0 (0,0)	2 (0,0)	1 (0,0)	
mos 45,X[66]/46,XY[96]	0 (0,0)	1 (0,0)	0 (0,0)	
45,X	7 (4,6)	119 (2,8)	5 (0,1)	
45,X,inv(10) (p15q11.2)	0 (0,0)	1 (0,0)	0 (0,0)	
mos45,X,-14[13]/46,XX,r(14) (p?10q?32)[7]	0 (0,0)	1 (0,0)	0 (0,0)	
45,XX,der (21; 22) (q10; q10) pat	0 (0,0)	2 (0,0)	0 (0,0)	
45,XX,der(13; 14) (q10; q10)mat	0 (0,0)	1 (0,0)	0 (0,0)	
45,XX,der(14; 21) (q10; q10)	0 (0,0)	0 (0,0)	1 (0,0)	
45,XY,der(13; 14) (q10:q10)	0 (0,0)	1 (0,0)	0 (0,0)	
45,XY,der(13; 14) (q10; q10)pat	0 (0,0)	1 (0,0)	0 (0,0)	
45,XY,der(13; 15) (q10; q10)	0 (0,0)	0 (0,0)	1 (0,0)	
45,XY,der(14; 21) (q10; q10)	0 (0,0)	1 (0,0)	0 (0,0)	
45,XY,der(5)t(5; 14) (q35; q13),-14	0 (0,0)	1 (0,0)	0 (0,0)	
45,XY,der(14; 21) (q10; q10)	0 (0,0)	1 (0,0)	0 (0,0)	
46,XX,t(1; 5) (q31; q3.3.3)	0 (0,0)	1 (0,0)	0 (0,0)	
46,XX,t(4; 9) (p14; q3.4.2) pat	0 (0,0)	1 (0,0)	0 (0,0)	
46,XX,del (4) (p1?20)	0 (0,0)	0 (0,0)	1 (0,0)	
46,XX,?inv(10) (q11.1q21.2)	0 (0,0)	2 (0,0)	0 (0,0)	
46,XX,+13,der(13; 14) (q10; q10)	0 (0,0)	1 (0,0)	0 (0,0)	
46,XX,+21,der(21; 21) (q10; q10)	0 (0,0)	1 (0,0)	1 (0,0)	
46,XX,del(13) (q?14q?31)dn	0 (0,0)	1 (0,0)	0 (0,0)	
46,XX,der(12)t(?2; 12) (p21; p?1.3.1)	0 (0,0)	1 (0,0)	0 (0,0)	
mos 46,XX,der(12)t(12; ?) (p?; ?)[8]/46,XX,[5]	0 (0,0)	1 (0,0)	0 (0,0)	
mos 46,XX,der(14; 21) (q10; q10),+21[2]/46,XX[13]	0 (0,0)	1 (0,0)	0 (0,0)	
46,XX,der(15)	0 (0,0)	1 (0,0)	0 (0,0)	
46,XX,der(18)t(6; 18) (p21.3; q21.3)mat	0 (0,0)	1 (0,0)	0 (0,0)	
mos 46,XX,der(4)t(4; ?) (q?; ?)[5]/46,XX[5]	0 (0,0)	1 (0,0)	4 (0,1)	
46,XX,der(5)t(1; 5)	0 (0,0)	1 (0,0)	0 (0,0)	
46,XX,der(9)	0 (0,0)	1 (0,0)	1 (0,0)	
46,XX,dup(12) (p13.33p13.1)dn	0 (0,0)	0 (0,0)	1 (0,0)	
46,XX,t(1; 10) (q25; q1.1.2)	0 (0,0)	1 (0,0)	0 (0,0)	
46,XX,t(1; 8) (q31; q?23)pat	0 (0,0)	0 (0,0)	1 (0,0)	
46,XX,t(1; 9) (q?21; q?13)	0 (0,0)	1 (0,0)	0 (0,0)	
46,XX,t(11; 22) (q23.3; q11.2)inh	0 (0,0)	1 (0,0)	0 (0,0)	
46,XX,t(11; 22) (q23; q11.2)	0 (0,0)	1 (0,0)	0 (0,0)	
46,XX,t(11; 22) (q23; q11.2)pat	0 (0,0)	1 (0,0)	0 (0,0)	
46,XX,t(3; 5) (q?12; q?11.2)dn	0 (0,0)	0 (0,0)	1 (0,0)	
46,XX,t(7; 14) (q11.1:p11.2)mat	0 (0,0)	3 (0,1)	1 (0,0)	
46,XX,t(7; 14) (q11.1; p11.2)inh	0 (0,0)	0 (0,0)	1 (0,0)	
46,XX,t(8; 15) (p11.2; q13)mat	0 (0,0)	1 (0,0)	0 (0,0)	
46,XX,t(8; 15) (p23; q24)mat	0 (0,0)	1 (0,0)	0 (0,0)	
46,XY,+13,der(13; 13) (q10; q10)	0 (0,0)	1 (0,0)	0 (0,0)	
46,XY,+13,der(13; 14) (q10; q10)	0 (0,0)	1 (0,0)	0 (0,0)	
46,XY,,der(14; 21) (q10; q10),+21	0 (0,0)	1 (0,0)	0 (0,0)	
46,XY,+21,der(21; 21) (q10; q10)	0 (0,0)	1 (0,0)	0 (0,0)	
mos 46,XY,add(1)[3]/46,XY[12]	0 (0,0)	1 (0,0)	0 (0,0)	
46,XY,add(18) (p?11)dn	1 (0,7)	0 (0,0)	0 (0,0)	
46,XY,del(13) (q32)	0 (0,0)	3 (0,1)	1 (0,0)	
46,XY,del(18) (?11.2)dn	0 (0,0)	0 (0,0)	1 (0,0)	
46,XY,der(17)	0 (0,0)	0 (0,0)	2 (0,1)	
46,XY,der(17)t(11; 17) (q13; q25)pat	0 (0,0)	1 (0,0)	0 (0,0)	
46,XY,der(17)t(17; 19) (q25; q13?3)inh	0 (0,0)	1 (0,0)	0 (0,0)	
46,XY,der(18)t(3; 18) (q25; q21.3)	0 (0,0)	1 (0,0)	0 (0,0)	
46,XY,der(21)t(14; 21) (q22; q2.2.1)mat	0 (0,0)	1 (0,0)	0 (0,0)	
46,XY,der(7)t(7; 17) (q32; p1.1.2)mat	0 (0,0)	1 (0,0)	0 (0,0)	
46,XY,der(8)t(8; 15) (p23; q24)mat	0 (0,0)	1 (0,0)	0 (0,0)	
46,XY,der(9)t(5; 9) (p13; p24)pat	0 (0,0)	1 (0,0)	0 (0,0)	
46,XY,inv(10) (p11q21)	0 (0,0)	0 (0,0)	1 (0,0)	
46,XY,inv(2) (p11.2q13)	0 (0,0)	0 (0,0)	1 (0,0)	
46,XY,inv(5) (p13q13)	0 (0,0)	3 (0,1)	1 (0,0)	
46,XY,inv(9) (p11q12)	0 (0,0)	13 (0,3)	15 (0,4)	
46,XY,inv(9) (p12q13)	0 (0,0)	14 (0,3)	13 (0,3)	
46,XY,r(13)	0 (0,0)	2 (0,0)	0 (0,0)	
46,XY,der(13; 13) (q10; q10)	0 (0,0)	1 (0,0)	0 (0,0)	
46,XY,t(11; 22) (q23.3; q11.2)	0 (0,0)	1 (0,0)	0 (0,0)	
46,XY,t(2; 9) (q33; q32)	0 (0,0)	1 (0,0)	0 (0,0)	
46,XY,t(3; 7) (q2?5.3; p13)pat	0 (0,0)	1 (0,0)	0 (0,0)	
46,XY,t(5; 13) (11.2; 12.1)	0 (0,0)	1 (0,0)	0 (0,0)	
46,XY,t(6; 8)	0 (0,0)	1 (0,0)	0 (0,0)	
46,XY,t(7; 8) (p21; q2?2)pat	0 (0,0)	0 (0,0)	1 (0,0)	
46,XY,t(8; 17) (q?24; ?25)	0 (0,0)	0 (0,0)	1 (0,0)	
mos 47,XX,+18[15]/46,XX [15]	0 (0,0)	1 (0,0)	0 (0,0)	
47,XXY	0 (0,0)	5 (0,1)	2 (0,1)	
47,XYY	0 (0,0)	3 (0,1)	2 (0,1)	
47,XX,+ inv dup(14) (q11.1	0 (0,0)	0 (0,0)	1 (0,0)	
47,XX,+13	0 (0,0)	15 (0,3)	13 (0,3)	
47,XX,+18	2 (1,3)	108 (2,5)	111 (3,0)	
47,XX,+18,del(18) (p?10)	0 (0,0)	0 (0,0)	1 (0,0)	
47,XX,+21	4 (2,6)	109 (2,5)	156 (4,2)	
47,XX,+21,inv(9) (p11q13)	0 (0,0)	2 (0,0)	0 (0,0)	
47,XX,+21/46,XX	0 (0,0)	1 (0,0)	0 (0,0)	
mos 47,XX,+21[10]/46,XX[6]	0 (0,0)	0 (0,0)	2 (0,1)	
mos 47,XX,+21[11]/46,XX[4]	0 (0,0)	0 (0,0)	1 (0,0)	
mos 47,XX,+21[13]/46,XX[15]	0 (0,0)	0 (0,0)	1 (0,0)	
mos 47,XX,+21[17]/46,XX[6]	0 (0,0)	1 (0,0)	0 (0,0)	
mos 47,XX,+21[4]/46,XX[10]	0 (0,0)	1 (0,0)	0 (0,0)	
47,XX,+22	0 (0,0)	2 (0,0)	0 (0,0)	
47,XX,+9	0 (0,0)	0 (0,0)	1 (0,0)	
47,XX,+der(22)t(11; 22) (q23.3; 11.2)mat	0 (0,0)	0 (0,0)	1 (0,0)	
47,XX,+der(9)	0 (0,0)	1 (0,0)	0 (0,0)	
mos 47,XX,+mar[15]/46,XX[15]	0 (0,0)	0 (0,0)	1 (0,0)	
mos 47,XX,+mar[4]/46,XX[21]	0 (0,0)	0 (0,0)	1 (0,0)	
47,XXX	0 (0,0)	9 (0,2)	2 (0,1)	
47,XY,+13	3 (2,0)	30 (0,7)	18 (0,5)	
47,XY,+13, inv(9) (p12q13)	0 (0,0)	1 (0,0)	0 (0,0)	
47,XY,+13,inv(9) (p12q13)	0 (0,0)	1 (0,0)	0 (0,0)	
47,XY,+18,inv(10) (p11.2q21.2)	0 (0,0)	1 (0,0)	0 (0,0)	
47,XY,+21	0 (0,0)	116 (2,7)	147 (4,0)	
47,XY,+21,inv(9) (p11q13)	0 (0,0)	1 (0,0)	0 (0,0)	
47,XY,+21[14]/46,XY[3]	0 (0,0)	0 (0,0)	1 (0,0)	
47,XY,+21[27]/46,XY[3]	0 (0,0)	1 (0,0)	0 (0,0)	
47,XY,+22	1 (0,7)	0 (0,0)	0 (0,0)	
47,XY,+9	0 (0,0)	3 (0,1)	0 (0,0)	
47,XY,+der(22)t(q23; q11.2)mat	0 (0,0)	1 (0,0)	0 (0,0)	
47,XY,+i(18) (q11.1)	0 (0,0)	0 (0,0)	1 (0,0)	
47,XY,+mar[14]/46,XY[6]	0 (0,0)	0 (0,0)	1 (0,0)	
47,XY,+mar[5]/46,XY[14]	0 (0,0)	1 (0,0)	0 (0,0)	
69,XXX	1 (0,7)	32 (0,7)	2 (0,1)	
69,XXY	0 (0,0)	24 (0,6)	3 (0,1)	

The rate of failed cell cultures was higher among the youngest group (5.2%) compared to the 20–34 group (2.0%) and the ≥35 group (1.2%, p < 0.001). Abnormal fetal ultrasound findings were the primary indication for invasive procedures across all age groups, particularly in adolescents (86.3%, Table 2). In older groups, advanced maternal age (7.2%) and high-risk first-trimester screening results became more prominent indications, with the latter rising from 13.1% in the ≤19 group to 35% in the ≥35 group.

The choice of procedure varied significantly by age (Table 2). CVS was more common in younger women, while amniocentesis was predominantly performed in women aged ≥35 years (p < 0.001). The distribution of fetal sex demonstrated no statistically significant variation across age groups; however, a higher proportion of cases with “unspecified fetal sex” (>5%) was documented in the adolescent cohort.

### Relative risk (RR) between groups

**FIGURE 2:**
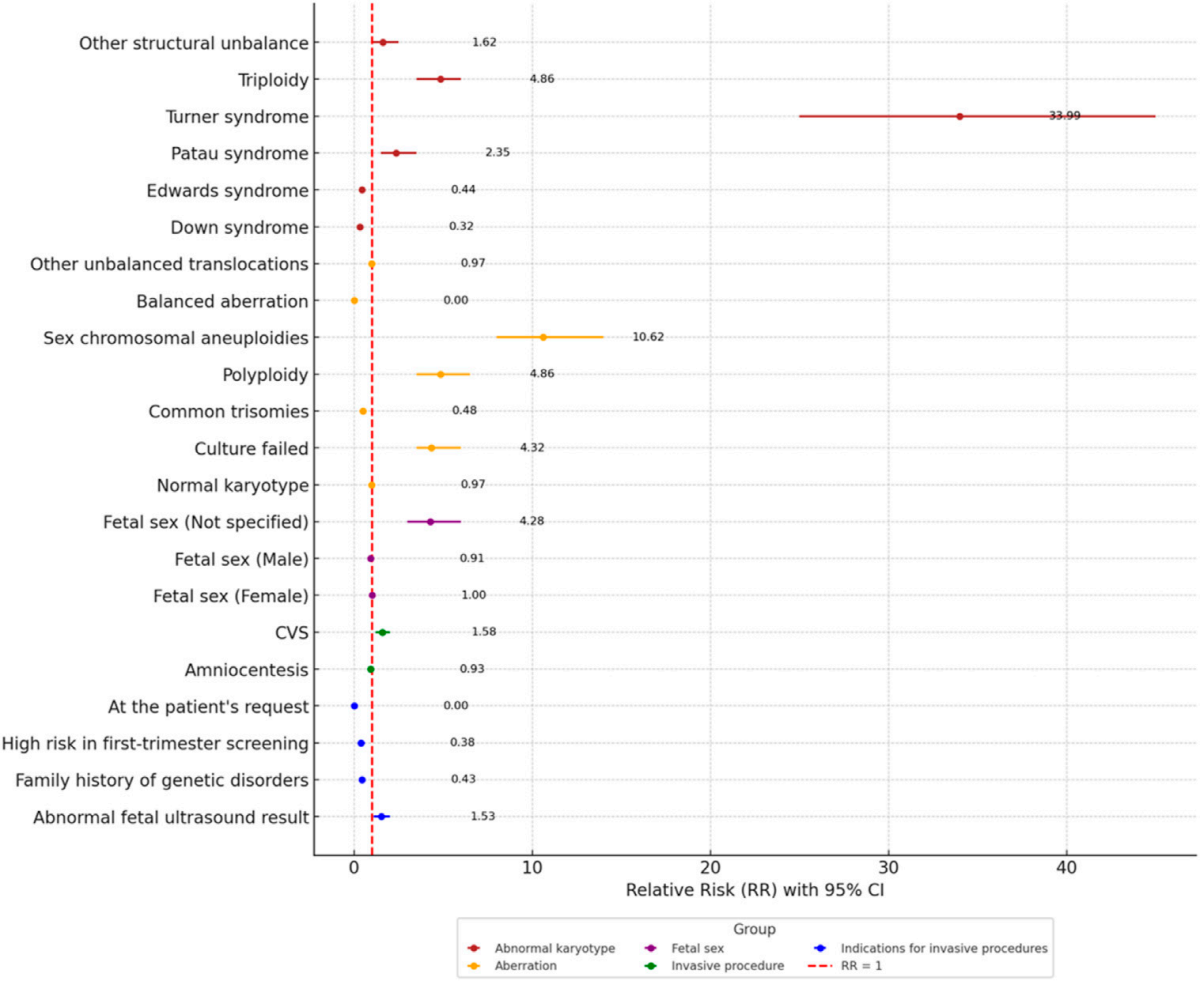
Forest plot for Relative Risk (RR) for qualitative variables between the age group ≤19 years and 20–34 years.

Relative risks (RR) were calculated to compare the outcomes between adolescents aged ≤19 years and women aged 20–34 years (Figure 2). Adolescents demonstrated a significantly higher risk of culture failure (RR = 2.58, 95% CI: 1.27–5.22), reflecting a 2.5-fold greater likelihood of missing karyotype results than the older group. They also showed a lower risk of “common trisomies” (RR = 0.63, 0.33–1.20), though the wide confidence interval limits the certainty of this finding. The risk of sex chromosome aneuploidies (RR = 1.34, 0.64–2.82), amniocentesis (RR = 0.99, 0.92–1.06), and CVS (RR = 1.06, 0.74–1.51) was comparable between the groups. However, adolescents were more likely to have cases of “unspecified fetal sex” (RR = 2.25, 1.16–4.35) and abnormal ultrasound findings (RR = 1.13, 1.05–1.20), while showing a lower incidence of high-risk screening test results (RR = 0.63, 0.42–0.95).

**FIGURE 3:**
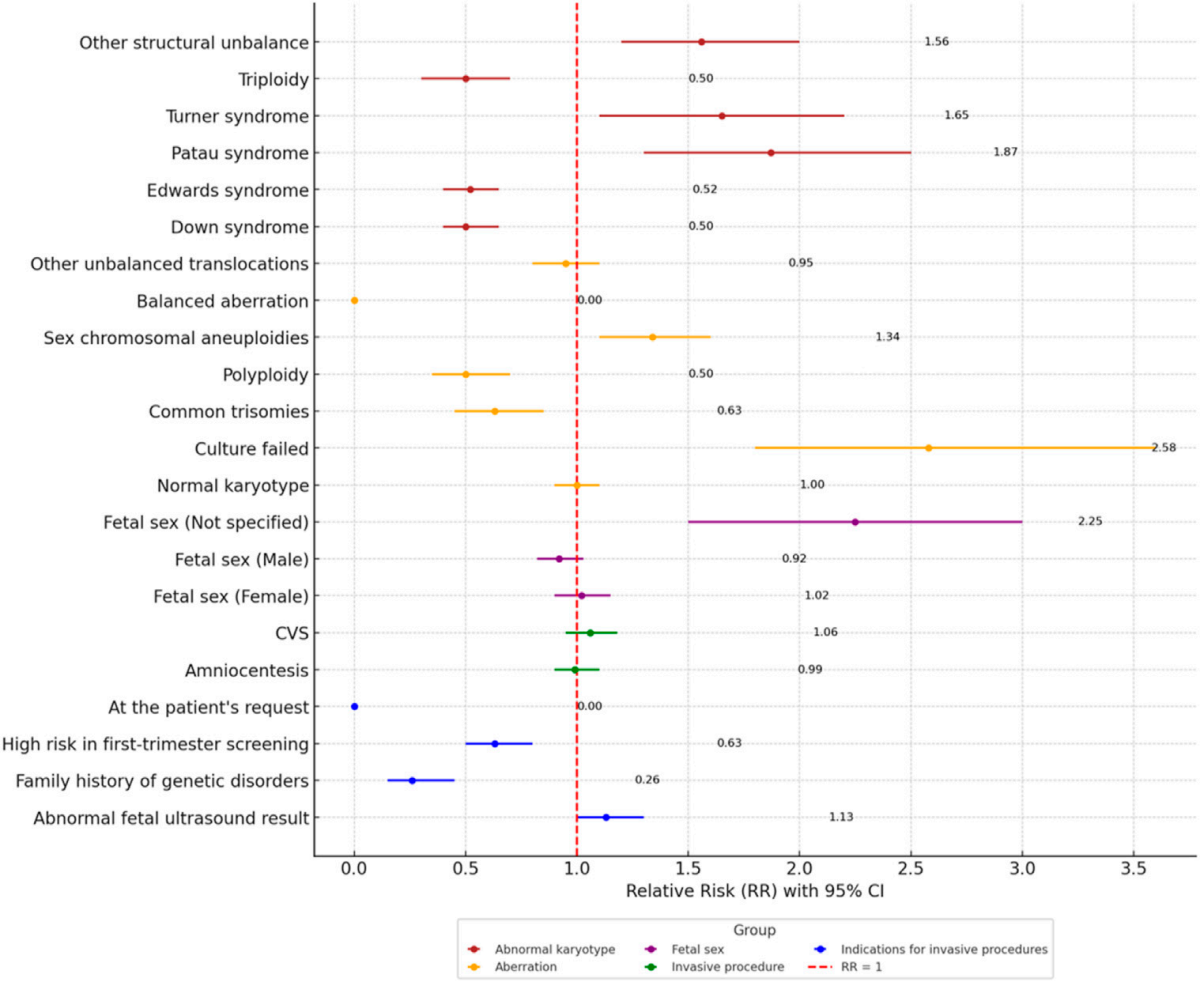
Forest plot of relative Risk (RR) for qualitative variables between the age group ≤19 years and ≥35 years.

In comparison with women aged ≥35 (Figure 3), the ≤19 groups exhibited an even higher disparity in certain areas. Adolescents had a nearly fourfold higher risk of culture failure (RR = 4.32, 2.07–9.00) and significantly lower risks of common trisomies (RR = 0.48, 0.26–0.92) and Down syndrome (RR = 0.32, 0.12–0.85), reinforcing the association of autosomal aneuploidies with advanced maternal age. Conversely, adolescents showed a much higher risk of sex chromosome aneuploidies (RR = 10.62, 4.44–25.44) and Turner syndrome (RR = 33.99, 10.91–105.89). Additionally, the risk of “unspecified fetal sex” was four times higher among adolescents (RR = 4.28, 2.15–8.54). Abnormal ultrasound findings were more prevalent in the ≤19 group (RR = 1.53, 1.43–1.64), while high-risk screening test results were less common (RR = 0.38, 0.25–0.57). The use of CVS was higher among adolescents (RR = 1.58, 1.10–2.27), whereas amniocentesis was performed at a similar rate (RR = 0.93, 0.86–1.00).

### Detection rates of abnormal karyotypes between groups

In the case of CVS, the percentage of abnormal karyotypes increases with age (30.8% in women ≤19 years, 41.4% in those aged 20–34, and 45.6% in those ≥35), whereas the number of procedures required to identify one abnormal result decreases accordingly (3.25, 2.42, and 2.19, respectively). This is favorable for older patients (≥35 years) because a higher proportion of abnormalities translates into fewer procedures per detected abnormal karyotype.

Conversely, for amniocentesis, the highest rate of abnormal karyotypes (15%) is observed in the youngest group (≤19 years), while the lowest (11.9%) occurs in patients ≥35 years. At the same time, the number of procedures per abnormal finding increases with age (from 6.68 to 8.44), implying that although older patients are more frequently eligible for amniocentesis, more procedures must be performed to detect a single abnormal result.

The combined data for both tests (CVS + amniocentesis) reveal the highest proportion of abnormal karyotypes (18%) and the lowest number of procedures per abnormal diagnosis (5.55) in the 20–34 age group. In contrast, the ≥35-year group shows the lowest proportion of abnormal karyotypes (15.5%), requiring 6.46 procedures for each confirmed abnormal finding.

**FIGURE 4:**
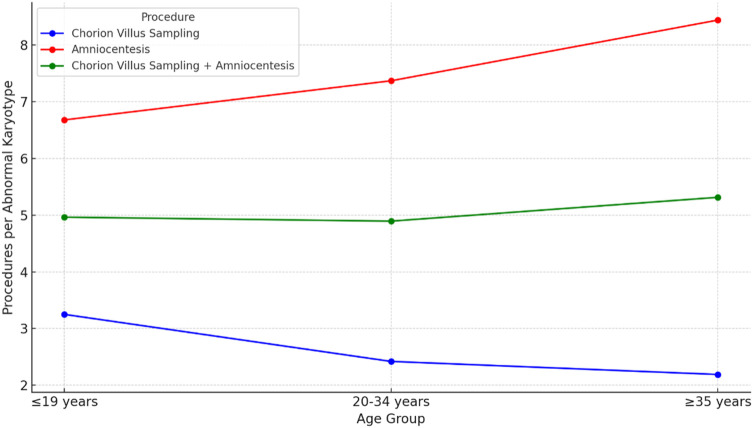
Detection rates of abnormal karyotypes and the number of procedures per abnormal karyotype.

Figure 4 presents the average number of procedures required to detect one abnormal karyotype, stratified by maternal age and type of invasive procedure.

## Discussion

**FIGURE 5:**
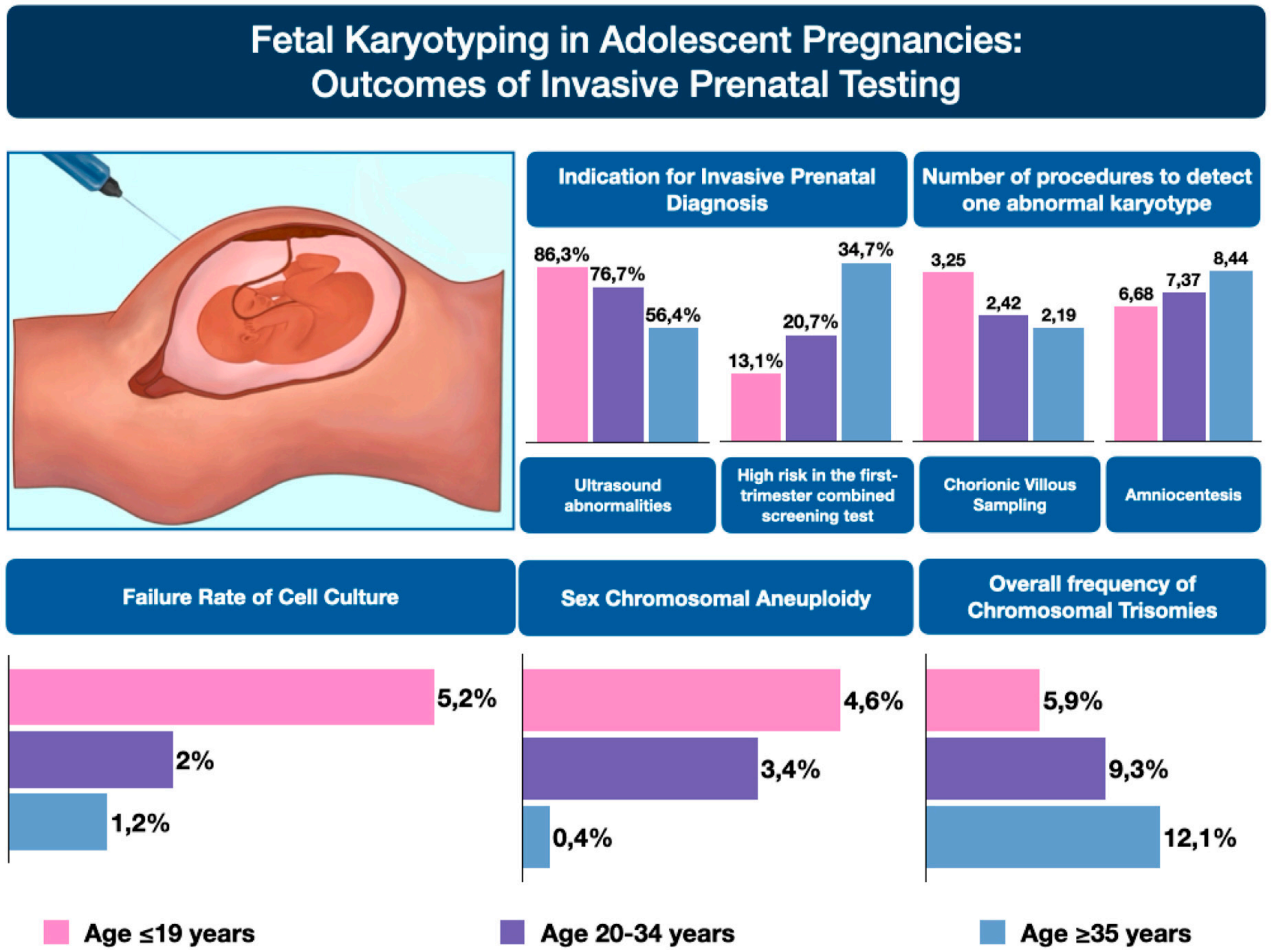
Central illustration.

Using retrospective cohort data from 8,155 pregnancies, we found that adolescent pregnancies (≤19 years) exhibit a distinct profile of chromosomal abnormalities, indications for invasive procedures, and diagnostic yield compared to older age groups. These findings warrant further investigation and discussion (Figure 5 – Central Illustration).

Our study suggests that younger patients may be at an increased risk of gonosomal aneuploidies, contrary to previous reports (Elmerdahl Frederiksen et al., 2024). It is essential to emphasize that prenatal fetal sex determination is crucial for women at high risk of severe sex-linked genetic disorders (Finning and Chitty, 2008). However, our study found that adolescents were more likely to present cases of “unspecified fetal sex” (RR = 2.25, 95% CI: 1.16–4.35) in fetal karyotyping. Moreover, the prevalence of gonosomal aneuploidies in the ≤19-year-old group was 4.6%, notably higher than in the 20–34-year-old group (3.4%) and significantly higher than in women aged ≥35 years (0.4%). This contrasts with the study by Elmerdahl Frederiksen et al., (2024), where no significant association between maternal age and the risk of monosomy X or other sex chromosome aneuploidies was observed. Regarding mosaic aneuploidies, our analysis revealed no cases of mosaic Turner syndrome or mosaic Down syndrome in patients ≤19 years, whereas these were detected in older age groups. Specifically, 8 cases (0.2%) of mosaic Turner syndrome were observed in the 20–34-year-old group, along with 5 cases (0.1%) in women aged ≥35 years. Similarly, mosaic Down syndrome was identified in 5 cases (0.1%) in both the 20–34 and ≥35-year-old groups. Elmerdahl Frederiksen et al., (2024) did not differentiate mosaic aneuploidies as a separate category, making our findings an essential contribution to the literature on the prevalence of these chromosomal abnormalities across different maternal age groups.

A higher prevalence of Turner syndrome (45,X) in adolescents supports the notion that, while autosomal trisomies are strongly associated with advanced maternal age, specific sex chromosome aneuploidies may be more common in very young mothers. In our study, the prevalence of Turner syndrome (45,X) was significantly higher among adolescent mothers (4.6%) compared to women aged 20–34 years (2.8%) and those aged ≥35 years (0.1%). These findings should be interpreted in light of previous studies like Hagman et al., who highlighted that advanced maternal age is a risk factor for giving birth to a girl with Turner syndrome (Hagman et al., 2010). On the other hand, Gravholt et al., (1996) have provided insights into the general epidemiology of Turner syndrome and its characteristics across age groups. Gravholt et al. also observed that only a subset of prenatally detected cases are confirmed postnatally, highlighting the diagnostic challenges, especially in mosaicism cases (Gravholt et al., 1996). This underscores a limitation of our study, as postnatal confirmation was unavailable. Our analysis suggests that the increased prevalence of Turner syndrome in adolescent mothers may result from greater diagnostic sensitivity to anomalies identified during prenatal ultrasounds in this group. Abnormal ultrasound findings were the primary indication for invasive testing in adolescents (86.3%), potentially explaining the higher detection rate of 45,X cases in this population. This is particularly relevant as Turner syndrome is associated with a higher incidence of congenital heart defects, often detected prenatally and accompanied by characteristic intrauterine findings, such as markedly increased nuchal translucency (NT) or hydrops (Surerus et al., 2003).

However, aside from these ascertainment factors, a genuine biological predisposition may also underlie the higher rate of 45,X observed in the youngest mothers. Recent evidence indicates that the relationship between maternal age and aneuploidy risk is U-shaped rather than linear. Gruhn et al. (2019) demonstrated that oocyte aneuploidy rates are elevated not only in women of advanced maternal age but also in adolescents, with the lowest incidence in the mid-20s (Gruhn et al., 2019). Whole-chromosome nondisjunction errors were particularly frequent in oocytes from the youngest patients, offering a potential explanation for the excess of 45,X conceptions in teenage pregnancies (Gruhn et al., 2019). Our findings of an increased Turner syndrome incidence in adolescents align with earlier reports by Kajii and Ohama (1979) and Warburton et al., (1980), who first described an inverse maternal age effect for monosomy X. Notably, Warburton et al. observed that monosomy X was disproportionately common among spontaneous abortions in very young women, a pattern not explained by low paternal age or gravidity (Warburton et al., 1980). In contrast, other studies have reported conflicting data, Hassold et al. (1980) found no significant association between maternal age and the incidence of sex chromosome monosomy, consistent with the findings of Elmerdahl Frederiksen et al. (2024) who noted no age-related difference in monosomy X risk in a sizeable prenatal cohort.

When analyzing trisomy prevalence, our data demonstrated a maternal age-related trend consistent with previous trisomy 21 and 18 studies but a divergent pattern for trisomy 13 (Cuckle and Morris, 2020). In the ≤19-year-old group, trisomy 21 prevalence was 2.6%, and trisomy 18 occurred in 1.3% of cases, lower than in older groups. However, trisomy 13 was more frequent in the ≤19-year-old group (2.0%) compared to the 20–34-year-old (1.0%) and ≥35-year-old groups (0.8%). This differs from Elmerdahl Frederiksen et al. (2024), Frederiksen et al. (2018) and Cuckle and Morris, (2020), who reported a consistent increase in trisomy prevalence with maternal age, suggesting that trisomy 13 may have a different age-related risk profile or be influenced by other contributing factors in younger patients.

Indeed, evidence from assisted reproduction indicates that even young couples can produce a notable proportion of aneuploid embryos. Preimplantation genetic testing for aneuploidy (PGT-A) studies have reported that in women under 35 years old, a substantial fraction of embryos are chromosomally abnormal. For instance, one analysis found that over 60% of embryos from patients younger than 35 were aneuploid, compared to approximately 72% in patients over 40 (Idarraga et al., 2022). Moreover, recent large-scale PGT-A data corroborate these observations. In a multi-center study analyzing tens of thousands of IVF embryos, nearly half of tested blastocysts in patients under 35 had whole-chromosome aneuploidies, with an additional 15%–18% classified as mosaic (containing both euploid and aneuploid cell lines) (Spinella et al., 2018). In line with this, a 2023 meta-analysis reported that mosaic embryos were more frequent in women <34 years than in those ≥34 years, despite overall aneuploidy rates increasing with age (Cascales et al., 2023). Common types of aneuploidy observed in embryos from young couples predominantly involve the smaller autosomes—particularly trisomies of chromosomes 15, 16, 21, and 22 — paralleling the patterns seen in spontaneous first-trimester miscarriages (Shahbazi et al., 2020). Sex chromosome abnormalities are also regularly identified in preimplantation embryos, illustrating that such meiotic segregation errors can arise independent of advanced maternal age (Gu et al., 2021).

These data underscore that younger maternal age does not equate to the absence of meiotic errors at conception. Thus, our observation of specific aneuploidies (such as 45,X and trisomy 13) in adolescent pregnancies is consistent with the notion that inherent chromosomal instability can occur even in early reproductive life, independent of advanced maternal age.

This highlights the crucial role of ultrasonography in detecting fetal anomalies and guiding clinical decision-making, particularly in younger pregnant patients. However, this may reflect a selection bias, as younger patients might be overrepresented in cases with more severe phenotypic abnormalities detectable by ultrasound.

The indication for invasive testing partially explains the observed differences in aneuploidy prevalence across age groups. In our cohort, as previously noted, the primary indication for invasive procedures in patients ≤19 years was abnormal ultrasound findings, whereas in older groups, high-risk first-trimester screening and advanced maternal age were more common. The greater reliance on ultrasound findings in adolescents, as opposed to first-trimester combined screening test results, aligns with our previous research, where we observed a higher incidence of abnormalities detected during ultrasound examinations in pregnancies among minors (Staniczek et al., 2025).

The success rate of culture outcomes also differed from the results. According to the literature, amniotic fluid culture failure occurs in approximately 0.44% of cases (Lam et al., 1998). While some studies suggest an association with chromosomal abnormalities (Reid et al., 1996), others found no significant correlation (Lam et al., 1998). This association likely stems from fetal anomalies, leading to decreased fetal cell shedding (Reid et al., 1996). Similarly, Lam et al., (1998) observed no significant difference in culture failure rates between normal and abnormal karyotypes but noted higher failure rates in advanced pregnancies with fetal structural defects. Our findings partly support this, as abnormal ultrasound results were the primary indication for invasive adolescent testing. The culture failure rate was significantly higher in adolescents (5.2%) than in women aged 20–34 years (2.0%) and ≥35 years (1.2%, p < 0.001), with a relative risk of approximately 2.5 times and over 4 times higher, respectively.

The results of our study demonstrate significant differences in the efficiency of invasive prenatal diagnostic procedures across age groups. The utilization of CVS in the adolescent group warrants discussion. While CVS offers the advantage of earlier diagnostic information, it has known limitations in accuracy and a greater likelihood of culture failure in certain cases. Thus, the decision to perform CVS in these young patients underscores how critical timely prenatal information was considered in managing adolescent pregnancies. Indeed, abnormal ultrasound findings were the leading indication for invasive testing in adolescents (86.3%), which explains the inclination toward earlier CVS rather than waiting for second-trimester amniocentesis. In our cohort, the decision to proceed with CVS was guided not only by the clinical indication but also by the technical feasibility of the procedure. In Poland, CVS is routinely performed using the transabdominal approach, which is generally considered safer and more acceptable for patients. Furthermore, the procedure was only undertaken following appropriate counseling and after obtaining informed consent from the patient and/or legal guardians.

For CVS, adolescents required 3.25 procedures to detect one abnormal karyotype, which was less efficient compared to women aged 20–34 years (2.42) and ≥35 years (2.19). These findings align with observations by Nicolaides et al. (1996), who reported that CVS, while offering the advantage of early diagnosis, has limitations in procedural accuracy, particularly in specific patient subgroups. This diagnostic inefficiency in younger women may stem from biological and technical challenges associated with CVS (Alfirevic et al., 2017). Similarly, Peuhkurinen et al. (2012) observed that first-trimester Down syndrome screening was less effective in women under 35 years of age, leading to an increased number of invasive procedures. Additionally, Bindra et al. (2002) reported that seven invasive procedures were required to detect one chromosomally abnormal fetus in their cohort. However, this study involved a highly selective population, with a median maternal age of 34 and a high proportion (47.1%) of women aged ≥ over 35. This underscores the importance of population-specific factors, such as maternal age distribution, when evaluating procedural efficiency. In contrast, amniocentesis demonstrated better diagnostic efficiency among adolescents, requiring 6.68 procedures per abnormal finding, compared to 7.37 in the 20–34 age group and 8.44 in the ≥35 group. These data suggest that the diagnostic method should be tailored to the patient’s age and the characteristics of the screened population, considering differences in efficiency and potential risks.

The strengths of our study include a diverse, large sample size of 8,155 pregnancies stratified by maternal age, making it the first study to focus specifically on adolescent pregnancies. The study addressed a population with limited chromosomal abnormalities and prenatal diagnostic efficacy data. Examining the types of chromosomal aberrations and comparing the outcomes of different invasive procedures provides new insights that could inform clinical practice and shape prenatal counseling, particularly in young mothers.

The limitations of this study should be acknowledged. Its retrospective design restricted the analysis to prenatal karyotyping and invasive procedure outcomes, as data were collected from outpatient centers where many patients underwent ultrasound examinations but were delivered elsewhere. The study population was predominantly white, which may limit the generalizability of findings to more diverse populations. Additionally, our study lacked data on the age of the fathers of the pregnant patients, as this information was not routinely recorded in our clinical database. This absence of paternal age data limits our ability to assess potential paternal contributions to fetal chromosomal abnormalities, including autosomal trisomies and Turner syndrome (45,X). Paternal factors can influence the occurrence of aneuploidies; for example, the missing X chromosome in 45,X Turner syndrome is of paternal origin in approximately 80% of cases (Idarraga et al., 2022; Bosch et al., 2001; Lorda-Sanchez et al., 1992). Moreover, advanced paternal age has been associated with increased frequencies of sex chromosome disomy in sperm (Chu et al., 2023). Without information on paternal age, we could not evaluate whether older paternal age or other paternal factors contributed to the chromosomal abnormalities observed, representing a notable gap that should be addressed in future studies.

Lastly, reliance on classical karyotyping, while excluding advanced techniques such as aCGH, may have resulted in the omission of structural or submicroscopic chromosomal aberrations, particularly in cases of cell culture failure. The exclusion of aCGH and techniques such as FISH was due to the fact that these methods were not reimbursed by the National Health Fund in Poland, and only a few patients in our database had undergone these tests. However, it is essential to note that even the use of the latest techniques may not overcome the limitation that some of these anomalies remain undetectable by newer molecular methods. Therefore, fetal karyotyping continues to be an essential component of prenatal diagnosis (Moczulska et al., 2023). Referral bias may also have influenced results, as younger patients were primarily referred for invasive procedures following abnormal ultrasound findings rather than high risk in the first-trimester combined screening test. Lastly, due to the study’s timeline, the absence of non-invasive prenatal testing (NIPT) data limits the applicability of findings in current clinical practices where NIPT plays a significant role. These limitations highlight areas for improvement in future research.

## Conclusion

In our study, we found that adolescent pregnancies (≤19 years) exhibit a distinct profile of chromosomal abnormalities, with a higher prevalence of Turner syndrome (45,X). Trisomy 13 was also more frequent in adolescents, deviating from the typical maternal age-related trends seen in trisomy 21 and 18. This higher occurrence may be linked to the greater reliance on ultrasound as the primary diagnostic trigger. Additionally, young mothers also experienced markedly higher culture failure rates and more frequent “unspecified fetal sex” results, reflecting the limitations of classical karyotyping in this group. The efficiency of invasive testing differed by age, with CVS being less yield-efficient in adolescents even though it was often utilized for early testing, whereas amniocentesis in adolescents had a comparatively better yield. Future practice and research should integrate advanced genomic techniques–for example, incorporating non-invasive prenatal testing and molecular karyotyping (aCGH microarray) – to enhance detection of chromosomal aberrations and reduce reliance on traditional karyotyping. Empowering prenatal diagnosis with such tools, in line with current guidelines, will improve accuracy and potentially obviate some invasive procedures in this high-risk young population.

## Data Availability

The data analyzed in this study was obtained as described in the Methods section. The following licenses/restrictions apply: the data contains information which are not publicly available due to privacy and ethical restrictions. Requests to access these datasets should be directed to Jakub Staniczek, jstaniczek@sum.edu.pl.
